# Direct Auger recombination and density-dependent hole diffusion in InN

**DOI:** 10.1038/s41598-018-22832-6

**Published:** 2018-03-15

**Authors:** Ramūnas Aleksiejūnas, Žydrūnas Podlipskas, Saulius Nargelas, Arūnas Kadys, Marek Kolenda, Kazimieras Nomeika, Jūras Mickevičius, Gintautas Tamulaitis

**Affiliations:** Institute of Photonics and Nanotechnology, Vilnius University, Saulėtekio ave. 3, Vilnius, LT-10257 Lithuania

## Abstract

Indium nitride has a good potential for infrared optoelectronics, yet it suffers from fast nonradiative recombination, the true origin of which has not been established with certainty. The diffusion length of free carriers at high densities is not well investigated either. Here, we study carrier recombination and diffusion using the light-induced transient grating technique in InN epilayers grown by pulsed MOCVD on c-plane sapphire. We show that direct Auger recombination governs the lifetime of carriers at densities above ~10^18^ cm^−3^. The measured Auger recombination coefficient is (8 ± 1) × 10^−29^ cm^−3^. At carrier densities above ~5 × 10^19^ cm^−3^, we observe the saturation of Auger recombination rate due to phase space filling. The diffusion coefficient of holes scales linearly with carrier density, increasing from 1 cm^2^/s in low-doped layers at low excitations and up to ~40 cm^2^/s at highest carrier densities. The resulting carrier diffusion length remains within 100–300 nm range, which is comparable to the light absorption depth. This feature is required for efficient carrier extraction in bipolar devices, thus suggesting MOCVD-grown InN as the material fit for photovoltaic and photonic applications.

## Introduction

Indium nitride with a direct band gap of 0.7 eV^1^ is an attractive material for infrared optoelectronics. However, InN layers of high quality are difficult to obtain. In addition to structural problems, InN suffers from high residual electron density (*n*_0_) caused by abundant point defects. *n*_0_ can be diminished by growing thick InN layers using molecular beam epitaxy (MBE)^2^, but this is an expensive and hardly scalable approach. Other growth techniques were also employed, including metalorganic chemical vapor deposition (MOCVD)^3^, chemical vapor deposition^4^, sputtering^5^, or even sol-gel spin coating^6^. The typical *n*_0_ values, however, remain in the range from 10^18^ cm^−3^ to mid-10^19^ cm^−3^. It is likely that InN-based devices will have to operate at high electron densities, thus, it is essential to understand the impact of high carrier density on carrier dynamics.

Carrier lifetime dependence on their density *τ*(*n*) is a powerful tool to reveal the dominating recombination mechanisms. Mainly linear or sublinear dependences were observed in InN layers by using the time-resolved photoluminescence, differential reflectance, or light-induced transient gratings (LITG) techniques. Based on these results, it was argued that Shockley-Read-Hall (SRH)^7–9^, Auger recombination in degenerate plasma^10^, or trap-assisted Auger recombination^11^ were the dominant recombination mechanisms in InN.

Carrier transport, especially that of minority holes, is less investigated. It was theoretically predicted that the room temperature hole mobility *μ*_h_ can reach 220 cm^2^/Vs in low-doped InN, but should drop rapidly with *n*_0_ above 10^17^ cm^−3 ^^12^. Experimentally, several techniques were used to measure *μ*_h_ at fixed hole density. *μ*_h_ = 17–36 cm^2^/Vs was estimated from sheet conductivity against sample thickness in Mg-doped layers at (1.4–3.0) × 10^18^ cm^−3 ^^13^. Variable magnetic field Hall measurements provided the mobility of heavy and light holes of 50 cm^2^/Vs and 600 cm^2^/Vs, respectively, in a sample with Mg doping at 3 × 10^20^ cm^−3 ^^14^. Hall measurements in InN layers Mg doped in a wide range from 10^18^ to 10^20^ cm^−3^ revealed p-type conductivity with similar *μ*_h_ of 20–30 cm^2^/Vs^15^. Application of LITG technique allowed for measuring the mobility of minority holes, which was ~40 cm^2^/Vs in high-quality MBE layers with *n*_0_ in the mid-10^17^ cm^−3 ^^16,17^.

This work is focused on the study of carrier dynamics in a wide range of carrier densities. Epilayers with different residual carrier densities were fabricated, while the increasing photoexcited carrier densities were generated using femtosecond laser pulses to ensure high time resolution. LITG technique is exploited to simultaneously extract the carrier lifetimes and their diffusion coefficients at different stages of the decay of nonequilibrium carrier density. The results enable the characterization of hole mobility and the estimation of diffusion lengths. The samples are grown by using MOCVD technique, which is most prospective for growing InN epilayers for photovoltaics and other applications on industrial scale. Optimized pulsed growth mode and temperature ramping were exploited to substantially increase the quality of the MOCVD-grown InN.

## Methods

A set of InN layers comprising 20 samples was grown using a close-coupled showerhead 3 × 2″ MOCVD reactor (Aixtron). All samples had the same structure: thin InN layers were deposited on top of 5-µm-thick c-plane GaN templates on sapphire. InN layers were grown at different temperatures from 570 to 630 °C, with the temperature either maintained constant or ramped up during the growth. Both continuous and pulsed delivery of precursors was exploited. A detailed description of the growth procedures and structural characterization of the layers is presented in ref.^18^. The layer thickness and the density of residual electrons in the epilayers varied from 30 nm to 400 nm and from 6 × 10^18^ cm^−3^ to 5 × 10^19^ cm^−3^, respectively. The electron density was determined by Hall measurements in van der Pauw geometry and confirmed by calculations of the Burstein-Moss shift of absorption edge and photoluminescence spectra observed in the samples.

The characterization of carrier dynamics has been performed by LITG technique^19^ at room temperature. The transient gratings were recorded by interference field of two coherent pulses formed by a holographic beam splitter. The 250-fs-duration pulses at 1030 nm were emitted by laser Pharos (Light Conversion) operating at 30 kHz rate. For probing, the pulses from optical parametric oscillator Orpheus (Light Conversion) tuned to 2350 nm were used. The samples were transparent at this wavelength, so the entire thickness of a sample was monitored in transmission geometry.

Carrier lifetime *τ* and ambipolar diffusion coefficient *D* at a certain delay time *t* were simultaneously obtained from LITG transients, i.e. the decay kinetics of diffraction efficiency *η*(*t*) recorded for several different grating periods *Λ*, according to the relation:

1 $$\frac{1}{{\tau }_{G}}=\frac{1}{\tau }+\frac{4{\pi }^{2}D}{{{\rm{\Lambda }}}^{2}},$$

where *τ*_G_ is the instantaneous decay time of LITG signal. *τ*_G_ is found from an exponential fit *η*(*t*) ∝ exp(−2*t*/*τ*_G_) at a certain delay *t*. *Λ* was varied by adjusting the angle between the interfering beams. Finally, residual (*n*_0_) and photoexcited (Δ*n*) carrier densities were controlled by using samples with different *n*_0_, and by changing the intensity of the recording pulses, respectively.

**Figure 1:**
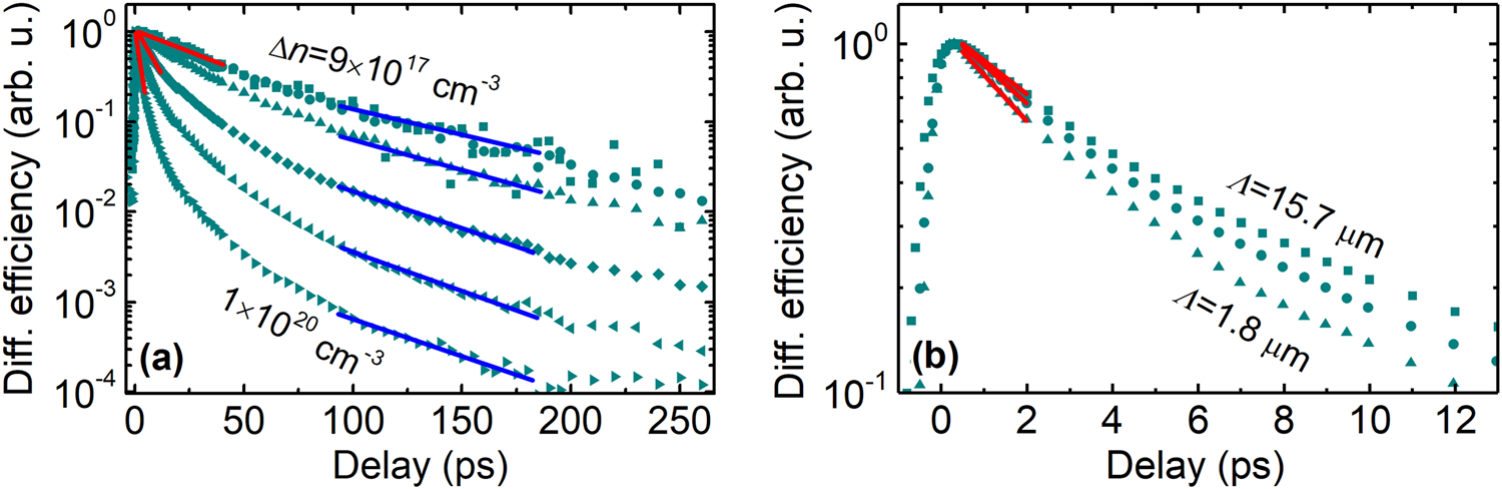
Normalized LITG transients recorded at (**a**) several excitation intensities or (**b**) three different grating periods at fixed excitation. Red and blue solid lines show exponential fits in the initial and tail parts of the transients, respectively.

Figure 1 shows the typical LITG transients recorded in one of the samples for different photoexcited carrier densities Δ*n* (a) and transient grating periods *Λ* (b). It can be seen that carrier decay becomes faster with increasing excitation intensity, thus, the instantaneous *τ*_G_ changes with delay as carriers recombine. As expected according to Eq. (1), *τ*_G_ becomes shorter for the transients measured at smaller *Λ*, due to the increasing influence of carrier diffusion^16,19^.

In this study, *τ*_G_ was determined either from the initial (<10 ps) or tail (>100 ps) part of the LITG transients. In the initial part, the photoexcited carrier density can be accurately estimated as Δ*n* = *αI*/*hν*, where *α* is the absorption coefficient, *I* is the excitation energy fluence (“excitation intensity”), and *hν* is the photon quantum energy; note that Δ*n* represents an averaged photoexcited carrier density over the period of transient grating at the very surface of a sample. This part of transient decay was used to study the dependences *τ*(*n*_0_ + Δ*n*) and *D*(*n*_0_ + Δ*n*). Assuming the equal densities of photoexcited electrons and holes, the total densities are *n* = *n*_0_ + Δ*n* for electrons and Δ*n* for holes. Recombination diminishes Δ*n* and condition *n*_0_ ≫ Δ*n* is achieved in the tail parts of the transients. This regime was used to study the dependence *τ*(*n*_0_).

## Results and Discussion

### Carrier recombination

**Figure 2:**
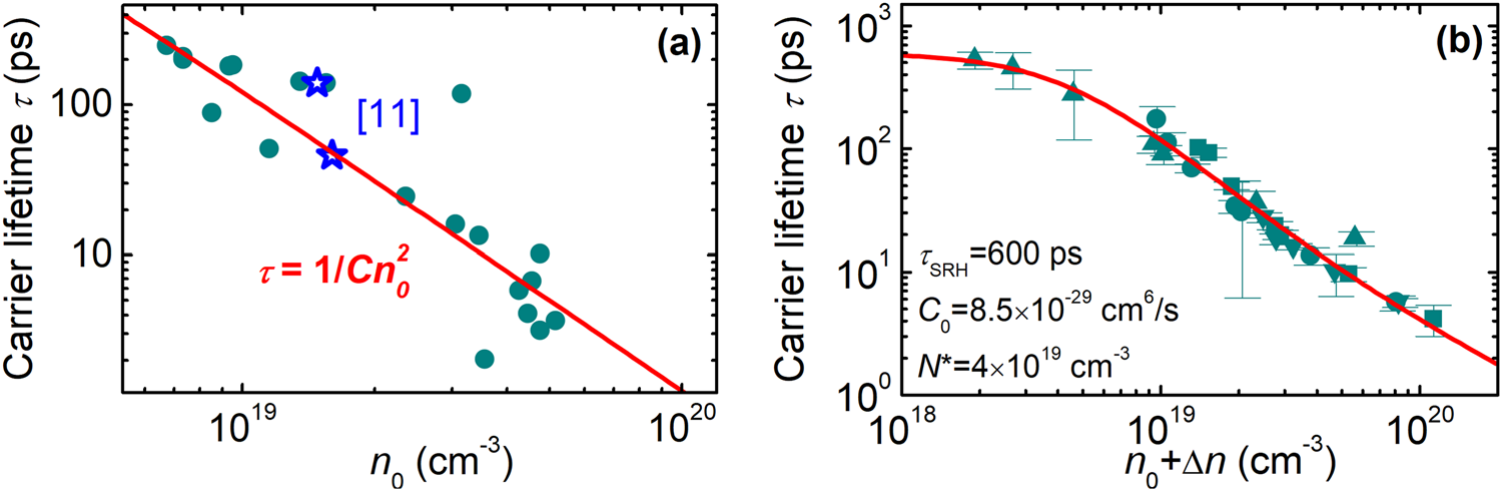
(**a**) Carrier lifetime *τ* as a function of residual electron density *n*_0_ in MOCVD (points) and MBE (stars) grown epilayers; solid line shows the fit *τ* = 1/*Cn*_0_^2^ at *C* = 8 × 10^−29^ cm^6^/s. (**b**) *τ* as a function of total carrier density *n*_0_ + Δ*n*; different symbols correspond to different samples. The solid line in (**b**) shows the fit according to Eq. (2) with parameters indicated.

Figure 2(a) shows carrier lifetime *τ* versus residual electron density *n*_0_ at low excitation regime (*n*_0_ ≫ Δ*n*); each point corresponds to a different sample. For comparison, we also provide the carrier lifetime reported in ref^11^ for MBE-grown epilayers of similar thickness. The lifetime in MOCVD samples varies from 2–3 ps to ~300 ps. Although these values are shorter than those measured in the best MBE layers (1.3 ns^20^ or even 5.4 ns^16^), they are, indeed, among the longest reported for MOCVD-grown layers^21^.

In Fig. 2(a), *τ* scales as 1/*n*_0_^2^, which is an indication of direct Auger recombination. The red solid line shows the function *τ* = 1/*Cn*_0_^2^ for *C* = 8 × 10^−29^ cm^6^/s. The obtained Auger coefficient value fits well to a general trend of Auger coefficients versus the band gap^22^. This result is in contrast to previously reported dependencies *τ* ∝ *n*^−1^ or *n*^−0.5 ^^7–9^. This discrepancy is most probably caused by ambiguities in the lifetime determination and total carrier density calibration in previous experiments. Photoluminescence and photoreflectivity techniques are sensitive to carrier diffusion into the sample’s depth, which is not the case in LITG experiments if carried out in transmission geometry^23^. Also, *τ* determination becomes uncertain because of a limited time resolution in time-resolved photoluminescence measurements at high densities and short delay times^24^, while the decay rate of differential transmission^7–9^ depends on the probe wavelength and does not always represent the rate of carrier recombination^25^. Meanwhile, in former LITG experiment^11^ reporting *τ* ∝ 1/B*n dependence (where B* was the effective recombination coefficient, accounting for radiative and non-radiative pathways), data from other studies was used to estimate *n*, making the calibration difficult.

To explore the carrier recombination in a wider density range, *τ* was measured at different excitation intensities in selected few samples. A 2.3 μm-thick MBE-grown InN epilayer was also included into this sample set to expand the *n* range towards lower densities. The *τ*(*n*) dependence is presented in Fig. 2(b). For densities between 8 × 10^18^ cm^−3^ and 5 × 10^19^ cm^−3^, *τ* decreases as *n*^−2^, but deviates from this dependence at lower and higher *n*. To describe *τ*(*n*) within the entire range, the standard ABC model was applied in the following form:

2 $$\frac{1}{\tau (n)}=\frac{1}{{\tau }_{SRH}}+C(n){n}^{2},$$

which takes into account the contributions of SRH and Auger recombination with characteristic times *τ*_SRH_ and 1/*Cn*^2^, respectively. Eq. (2) neglects the term of bimolecular radiative recombination, which should be slow on the time scale under study. Moreover, the rate of Auger recombination tends to saturate with increasing density due to phase-state filling, as was demonstrated in several semiconductors^26^ including InN^10^. This is taken into account by the following expression^27^:

3 $$C(n)=\frac{{C}_{0}}{(1+\frac{n}{{N}^{\ast }})},$$

where *C*_0_ is the density-independent Auger coefficient, and N^*^ is a constant characteristic carrier density. A good fit (red line in Fig. 2(b)) to experimental results was obtained using Eqs (2) and (3) and the parameters indicated in Fig. 2(b) for all samples.

### Carrier diffusion

**Figure 3:**
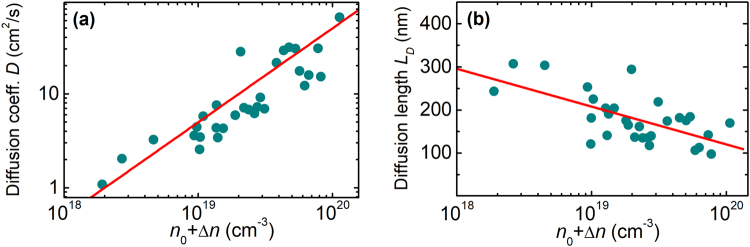
Experimental dependences of diffusion coefficient *D* (**a**) and diffusion length *L*_D_ (**b**) on total carrier density (points); lines are guides for the eye.

Figure 3 shows the ambipolar diffusion coefficient *D* (a) and the diffusion length *L*_D_ = (*τD*)^1/2^ (b) as functions of *n* = *n*_0_ + Δ*n* in the samples under study. The data in Fig. 3(a) is presented for the same samples as in Fig. 2(b). We note that the measured *D* values represent an averaged result over the period of transient grating. Therefore, the real diffusion coefficient could be even larger for those points where Δ*n* ≫ *n*_0_. The ambipolar diffusion coefficient is related to the monopolar electron (hole) diffusion coefficients *D*_e(h)_ by:

4 $$D=\frac{n+p}{\frac{p}{{D}_{e}}+\frac{n}{{D}_{h}}},$$

where *n* and *p* are the total electron and hole densities, respectively. According to Eq. (4) for *n* ≫ *p*, *D* ≈ *D*_h_, i.e., the ambipolar diffusion coefficient is determined by the diffusion of minority carriers. In InN under study, this condition is well satisfied for densities below 10^19^ cm^−3^. At higher excitations, *D*_h_ starts deviate from *D*, but the difference does not exceed 40% even at the highest *n*.

Approximately linear dependence of *D* on the total electron density can be traced in a wide range from 1 cm^2^/s at densities of 2 × 10^18^ cm^−3^ up to 40–50 cm^2^/s at ~10^20^ cm^−3^. Such a high *D* value cannot be explained by the simplified Einstein relation *D* = *μkT*/*e*, where *μ* is the mobility, *k* is the Boltzmann constant, *T* is the temperature, and *e* is the elementary charge. The previously reported mobilities for heavy and light holes of 50 and 600 cm^2^/Vs^14^ correspond to nondegenerate diffusion coefficients of 1.25 cm^2^/s and 15 cm^2^/s, respectively. Considerably higher *D*_h_ values can be expected due to carrier transport in degenerate and non-thermalized carrier plasma. For degenerate carriers, the diffusion coefficient is related to the carrier mobility by generalized Einstein relation^28^:

5 $${D}_{e(h)}=\frac{kT}{e}{\mu }_{e(h)}\frac{{F}_{1/2}}{{F}_{-1/2}},$$

where *F*_i_ are the Fermi-Dirac integrals that account for the increase of diffusivity in degenerate plasma, and *μ*_e(h)_ is the mobility of electrons (holes). Our calculations show that the degeneracy can account for the increase of diffusion coefficient from low density value of 2 cm^2^/s up to 20 cm^2^/s at 10^20^ cm^−3^, even for a constant carrier mobility. On the other hand, very high *D* values were obtained when carrier lifetime was only several picoseconds. At such short times, the transport of non-thermalized carriers might be expected^8,29^ due to considerable contribution of the light holes with a large non-degenerate diffusion coefficient. Moreover, ballistic-like spreading of short-living holes dragged by more mobile electrons might be also important for the enhancement of the effective hole diffusion coefficient within the initial few picoseconds after short-pulse excitation, similar to the phenomenon causing the velocity overshoot in low-temperature GaAs^30^.

The key parameter determining the responsivity of a photodiode is the diffusion length depending on the product of carrier lifetime and diffusion coefficient: *L*_D_ = (*τD*)^1/2^. Usually, a decrease in lifetime results in a drop of *L*_D_. In InN, the drop in *τ* with *n* due to increasing Auger recombination rate is compensated by a substantial increase in *D*. Therefore, as it is illustrated in Fig. 3(b), *L*_D_ exhibits only a slight decrease with growing carrier density and remains within 300–100 nm. The diffusion length of ~100 nm is comparable to the absorption depth for the light with photon energy above the band gap. Therefore, the 100–200 nm thick MOCVD-grown InN layers can be successfully exploited in photovoltaic applications.

In summary, the longest carrier lifetimes in InN epilayers grown by pulsed MOCVD at optimized conditions reach 300 ps, which is comparable to MBE-grown epilayers of similar thickness. Direct Auger recombination is shown to be the dominant nonradiative recombination mechanism at elevated carrier densities. Above 5 × 10^19^ cm^−3^, phase space filling causes the gradual saturation of the Auger recombination rate. The Auger recombination coefficient was found to be *C* = (8 ± 1) × 10^−29^ cm^6^/s. The ambipolar diffusion coefficient, which is equal or close to the hole diffusion coefficient, scales linearly with the carrier density and varies from 1 cm^2^/s to ~40 cm^2^/s. The high diffusion coefficient values revealed in InN epilayers are attributed to the cumulative effect of hole degeneracy and fast transport of nonthermalized light holes. The substantial increase in the diffusion coefficient at elevated carrier densities compensates the decrease in carrier lifetime, so that the diffusion length changes only unsubstantially within the range of 100–300 nm, what is of importance for application of InN in photovoltaics.

### Data availability

All data analyzed during this study are included in this published article.
